# Valorization of *Cladophora glomerata* Biomass and Obtained Bioproducts into Biostimulants of Plant Growth and as Sorbents (Biosorbents) of Metal Ions

**DOI:** 10.3390/molecules26226917

**Published:** 2021-11-16

**Authors:** Katarzyna Dziergowska, Maja Wełna, Anna Szymczycha-Madeja, Jacek Chęcmanowski, Izabela Michalak

**Affiliations:** 1 Department of Advanced Material Technologies, Faculty of Chemistry, Wrocław University of Science and Technology, Smoluchowskiego 25, 50-372 Wrocław, Poland; katarzyna.dziergowska@pwr.edu.pl (K.D.); jacek.checmanowski@pwr.edu.pl (J.C.); 2 Department of Analytical Chemistry and Chemical Metallurgy, Faculty of Chemistry, Wrocław University of Science and Technology, Wybrzeze Wyspianskiego 27, 50-370 Wrocław, Poland; maja.welna@pwr.edu.pl (M.W.); anna.szymczycha-madeja@pwr.edu.pl (A.S.-M.)

**Keywords:** macroalga, *Cladophora glomerata*, ultrasound assisted extraction, algal extract, nanoparticles biosynthesis, ZnO nanoparticles, germination tests, biostimulants of plant growth, *Raphanus sativus*, sorption of Cr(III) ions

## Abstract

The aim of this study was to propose a complete approach for macroalgae biomass valorization into products useful for sustainable agriculture and environmental protection. In the first stage, the effects of macroalgal extracts and ZnO NPs (zinc oxide nanoparticles) on the germination and growth of radish were examined. Macroalgal extract was produced from freshwater macroalga, i.e., *Cladophora glomerata* by ultrasound assisted extraction (UAE). The extract was used to biosynthesize zinc oxide nanoparticles. In germination tests, extracts and solutions of ZnO NPs were applied on paper substrate before sowing. In the second stage, sorption properties of macroalga, post-extraction residue, and ZnO NPs to absorb Cr(III) ions were examined. In the germination tests, the highest values of hypocotyl length (the edible part of radish), i.e., 3.3 and 2.6 cm were obtained for 60 and 80% extract (among the tested concentrations 20, 40, 60, 80, and 100%) and 10 and 50 mg/L NPs, respectively. The highest sorption capacity of Cr(III) ions (344.8 mg/g) was obtained by both macroalga and post-extraction residue at a pH of 5 and initial Cr(III) ions concentration of 200 mg/L. This study proves that macroalgae and products based on them can be applied in both sustainable agriculture and wastewater treatment.

## 1. Introduction

Radish (*Raphanus sativus*) belongs to the family *Brassicaceae*. It is frequently used as a model plant in germination tests because of its high global consumption (due to an abundance of vitamins, soluble sugar, and folic acid) as well as considerable adaptability [1]. Therefore, radish is often chosen to study the impact of biostimulants on plant growth and fertilizers on the development of below-ground vegetables [2].

Algal extracts can be used as natural plant growth stimulants in sustainable and organic crop production [3]. *Cladophora glomerata* is a green macroalga, which occurs both in marine and freshwater. It belongs to one of the most frequent seaweeds in the Polish water ecosystems [4]. It is considered to be harmful, as it occurs mainly in eutrophic water reservoirs and it can show a significant increase in daily biomass concentration, i.e., up to three times increase of biomass per day [5]. Although it is considered to be an environmental problem, *C. glomerata* can be used in various applications, due to its abundance of carotenoids; minerals; vitamins A, C, E, B_1_ and B_2_; amino acids and proteins; enzymes; fatty acids; sterols; terpenes; carbohydrates; glycosides; volatiles; and polyphenols [6,7]. It can be used, for example, in the cosmetic and food industries [8], agriculture [9], medicine and pharmacology [10], and as a bioaccumulator/bioindicator of environmental pollution [7].

Recently, more and more studies have shown the positive effect of macroalgal extracts on plant growth, including radish, for example, *Sargassum vulgare* (brown macroalgae) [3], *Codium taylorii* (green macroalgae), and *Pterocladia capillacea* (red macroalgae) [11]. Macroalgae, in the form of an extract or as a dry biomass, can influence radish growth, which has been proven in the case of *Ulva fasciata* (green) or *Sargassum lacerifolium* (brown) [12]. However, there are limited studies on the impact of *C. glomerata* extract on radish (e.g., [13]).

The same macroalgae that are used as biostimulants in sustainable agriculture can also be used as biosorbents of metal ions in wastewater treatment. Biosorption is a passive process that removes potentially toxic elements through different mechanisms. The me-chanism of metal ion absorption by biomass is complicated and involves different processes, such as adsorption, chelation/complexation, ion exchange, and surface precipitation [14,15]. Biosorption processes can be influenced by different factors, such as pH [16,17,18,19], temperature [15], initial metal ion concentration [20], contact time [17,19], competing ions/co-ions [15], and biosorbent dosage [18]. An ideal biosorbent should possess features such as availability, non-toxicity, high metal binding capacity, large-scale usability, and regeneration/re-usability. Different non-living biomasses have been reported for biosorption purposes, such as algal biomass, fungi, bacteria, and agricultural waste [14,15]. Moreover, for the sorption of heavy metals, metal and metal-oxide NPs, both biosynthesized with natural extracts, can also be used as a new generation of sorbents, for example, silver NPs [21] or zinc oxide NPs [22].

Algal biomass is considered to be the most employed biosorbent as compared with any other materials, because of its cell wall characteristics [15]. The macromolecules in an algal cell wall (e.g., polysaccharides) contain several functional groups (carboxyl, hydroxyl, amino, and sulfate) that can act as binding sites for pollutants [7]. Different types of algae have been reported to be biosorbents of metal ions (e.g., Pb, Hg, Zn, Cr, Ni, Co Cd, Cu, Sn, and As), among them green macroalgae [15], including *Cladophora* sp. to adsorb Cr(VI) [23] Cu(II), and Pb(II) ions [24]; *C. glomerata* to adsorb Cr(VI) ions [25]; *C. sericea* to adsorb Co(II), Cu(II), and Zn(II) ions [26]; *C. fascicularis* to adsorb Cu(II) and Pb(II) ions [27,28]; and *C. hutchinsiae* to adsorb Se(IV) [29] and U(VI) ions [30]. Moreover, dried *C. glomerata* biomass can be used as a biosorbent of heavy metal ions, and for products obtained from these macroalgae, for example, biochar in the pyrolysis [31] or even residues from the extraction of active compounds from algae [32]. According to the circular economy concept, using post-extraction biomass as a biosorbent minimizes waste gene-ration and enables the recovery of materials for other purposes [32,33,34,35].

Macroalgal extracts can also be used for the biosynthesis of metal or metal-oxide NPs, which can play roles as biostimulants of plant growth and biosorbents. These nanoparticles can be synthesized in three ways: physical, chemical, and biological. Currently, biosynthesis of NPs using plant extracts, fungi, and bacteria is very popular, but algal extracts are increasing in popularity. Examples of macroalgae used for the biosynthesis of ZnO NPs are green macroalgae *Halimeda tuna* [36] and *Caulerpa peltate* [37]; brown macroalgae *Sargassum muticum* [38,39], *Sargassum myriocystum* [37], and *Sargassum wightii* [40]; and red macroalgae *Hypnea valencia* [37] and *Gracilaria edulis* [41].

Recently, the use of metal and metal-oxide nanoparticles in agriculture has become more popular. According to Liu et al. [42], the following NPs have been most widely studied: gold, silver, aluminum, iron, titanium oxide, zinc oxide, aluminum oxide, iron (III) oxide, nickel oxide, copper oxide, and cerium oxide. Due to their smaller size and high surface area, metal NPs, as compared with their bulk parent materials, have enhanced physicochemical properties and biological activities. Owing to these properties, even small doses of NPs can affect seed germination and plant growth. They can influence parameters such as root elongation, plant growth, chlorophyll content, photosynthetic acti-vity, and resistance to oxidative stress. Moreover, some metal and metal-oxide NPs, inclu-ding ZnO NPs, have shown antifungal [43] or antibacterial activity [44]. ZnO NPs also play an important role in the production of many hormones, especially auxin, that are responsible for plant growth and yield [45].

In addition to improving seed germination and plant growth parameters, NPs also have the ability to adsorb heavy metal ions. Studies have shown that Zn NPs can successfully be used as sorbents for removing Zn(II), Hg(II) [46], Cd(II) [46,47], Cr(VI) [48], Ni(II) [22], Co [47], and Pb(II) ions [24,47] from aqueous solutions.

**Figure 1:**
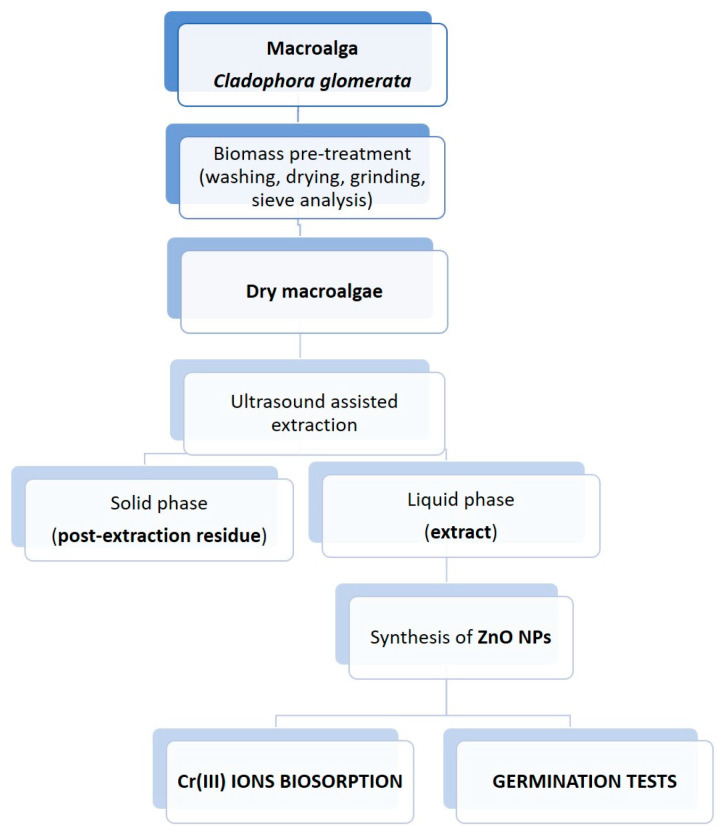
The general scheme of the proposed approach for the valorization of *Cladophora glomerata*.

The aim of this study was to propose a method for valorization of *C. glomerata*, which occurs in very large quantities in Poland. This biomass was used to obtain extract that could be used as a biostimulant of plant growth. Following the rules of a circular bioeco- nomy, the post-extraction residues produced during extraction were applied as biosorbents of metal ions. Moreover, the algal extract was used to biosynthesize ZnO NPs, which could be implemented both in sustainable agriculture and wastewater treatment. The general scheme of the proposed approach for valorization of *Cladophora glomerata* is presented in Figure 1.

## 2. Results and Discussion

### 2.1. Characteristics of Cladophora glomerata and Macroalgal-Based Products

The obtained macroalgal extract was a dark green-olive color. The average pH value of 100% extract was 7.33 ± 0.02, which was similar to the pH value of *C. glomerata* extract obtained in another study, i.e., 7.5 (also for ultrasound-assisted extraction) [49]. The multielemental composition of raw algal biomass, algal extract, and post-extraction residue is presented in Table 1. This freshwater macroalga contains mainly Ca, K, S, Mg, and P. This corresponds to the multielemental composition of the various *Cladophora* species presented in the review by Michalak and Messyasz [7]. The examined algal biomass did not contain toxic metal ions such as Cd, Hg, and Pb (which were below the limit of detection), but As was present. According to the literature, in many genera of algae, arsenic occurs in an organic form as arsenosugar, which is not acutely toxic, but its presence should be kept in mind [50]. Ultrasound-assisted extraction enabled extraction mainly of K, Na, Cu, P, S, and Mg. The post-extraction residue still contains valuable micro- and macroelements, and therefore can be used as a soil improver [35] or as a sorbent of metal ions [32,33,34,35]. This approach can reduce the costs of residue disposal [34].

The examined *Cladophora glomerata* extract also contained polyphenols; the total phenolic content was 73.5 ± 8.8 mg GAE/L (expressed as gallic acid equivalent)/L) of extract. Polyphenols are recognized as potent antioxidants, because of their capacity to donate ele-ctrons or hydrogen atoms and their stable radical intermediates [6,7]. *Cladophora* extracts, due to the content of phenolics, can also exhibit strong DPPH radical scavenging activity, i.e., the ability to neutralize free radicals [7]. The antioxidant activity of the *C. glomerata* extract was 0.0305 ± 0.0117 mmol/L of extract (expressed as Trolox equivalent). The percentage of the scavenging effect (inhibition of free radicals) by algal extract was 10.8 ± 0.9%.

### 2.2. Characteristics of ZnO NPs

The use of plant/algal extract for the biosynthesis of metal nanoparticles is considered to be an environmentally acceptable, clean and nontoxic “green procedure” [37,45]. The biologically active compounds in algal extracts can be responsible for the reduction and stabilization of zinc oxide nanoparticles, for example, fucoidan (pigment) from *Sargassum myriocystum* [37], phytochemicals such as polyphenols from plant extracts [51] or seaweeds, i.e., *Sargassum wightii* [40], and polysaccharides from *Sargassum muticum* [38].

The obtained dried ZnO NPs were in the form of a white powder. The average weight of the NPs obtained from four replications was 0.805 ± 0.045 g. The multielemental compositions of ZnO NPs synthesized from zinc sulphate heptahydrate and *C. glomerata* extract are presented in Table 1. An ICP-OES analysis confirmed the high content of Zn in NPs, as well as other ions such as Na and S which were incorporated into nanoparticles during biosynthesis.

**Figure 2:**
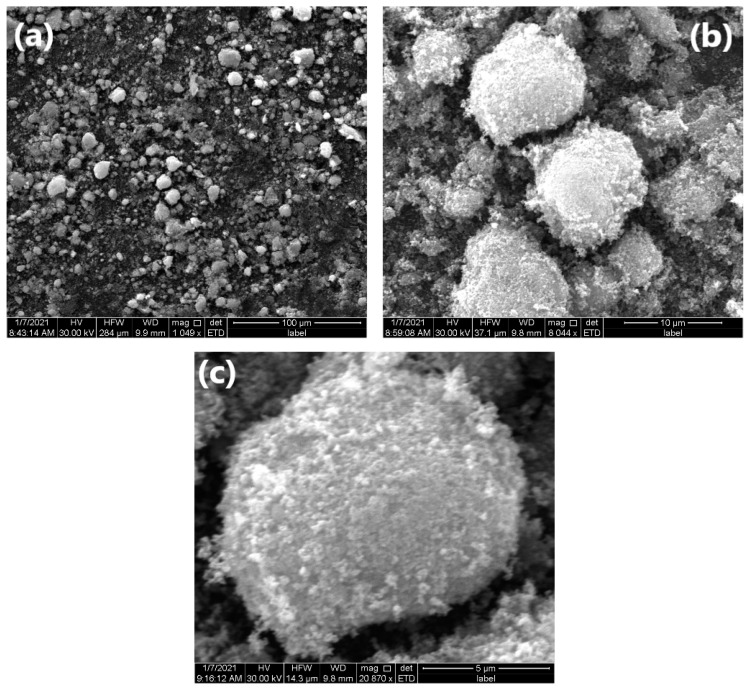
SEM images of ZnO NPs biosynthesized from *C. glomerata* extract—(**a**) magnification ~1000 times, (**b**) magnification ~8000 times, (**c**) magnification ~20,000 times.

The surface morphology of the resulting white powder was examined using a scanning electron microscope. The SEM analysis revealed the surface morphology of ZnO NPs and allowed us to estimate the obtained structural shape. The obtained zinc oxide nanoparticles in the biosynthesis with *Cladophora glomerata* extract after drying are shown in Figure 2. As a result of biosynthesis, agglomerates of ZnO particles of various shapes and sizes were obtained (Figure 2a). On the one hand, the analyzed material is dominated by small (up to 5 µm) irregular shapes (Figure 2b). On the other hand, the spherical shape with a developed surface shows agglomerates with a size of approximately 10 µm, in which no cracks were found, and the degree of visible pores was small (Figure 2c). The surface of this powder consists of many fine ZnO agglomerates, the size of which can be estimated to be 0.1–0.2 µm (Figure 2). Similar to the research results of Fakhari et al. [51], spherical NPs were formed which grew and accumulated to form flower-shaped bundles. According to Elumalai and Velmurugan [52], the probable reason for aggregation is the high surface energy of ZnO NPs that usually occurs when synthesis is carried out in an aqueous medium and the densification results in narrow spaces between particles.

**Figure 3:**
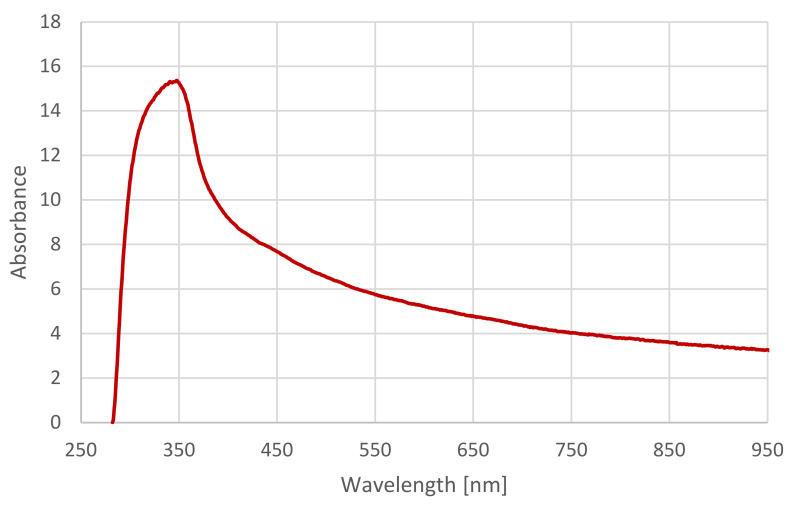
The UV-Vis spectrum of ZnO NPs biosynthesized from *C. glomerata* extract.

The UV-Vis spectrophotometric analysis of synthesized ZnO NPs is shown in Figure 3. As compared with the ZnSO_4_ solution, the UV-Vis of ZnO NPs had an absorption peak at 348 nm. The distinct absorption peak centered around 350 nm is specific for ZnO NPs due to their large excitation binding energy at room temperature [51]. Other researchers have also observed a peak at similar wavelengths in the UV-Vis of ZnO NPs (i.e., Vaishnaw et al. [53] (351 nm), Elumalai and Velmurugan [52] (370 nm), Itroutwar et al. [54] (377 nm), Fakhari et al. [51] (385 nm), as well as Senthilkumar and Thirumal [55] (325 nm)).

### 2.3. Germination Tests of Raphanus sativus

#### 2.3.1. Stimulation of Radish Growth by Macroalgal Extracts

**Figure 4:**
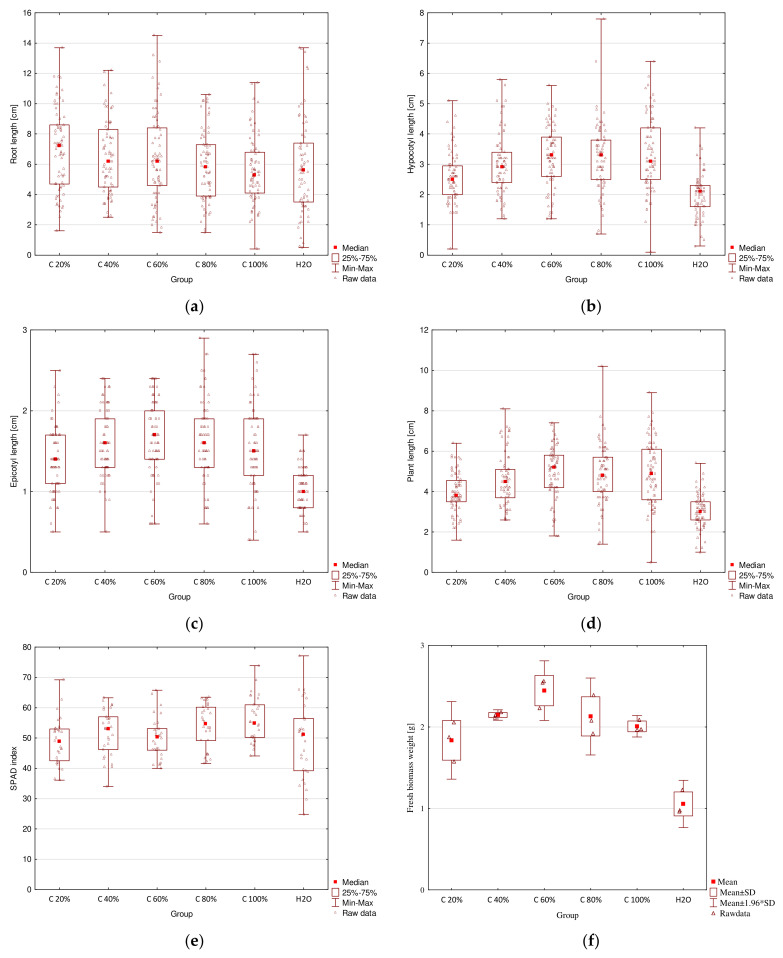
Box plots for results of germination tests with the use of various concentrations of *C. glomerata* extracts: (**a**) root length; (**b**) hypocotyl length; (**c**) epicotyl length; (**d**) whole plant length; (**e**) chlorophyll content; (**f**) fresh biomass weight.

The results for plant length, chlorophyll content, and fresh biomass weight measurements are presented in Table 2. The comparison of all the results is presented in box plots in Figure 4. *C. glomerata* extracts and ZnO NPs both showed a positive impact on seedling growth for some concentrations. They influenced root length, hypocotyl length, whole plant length, chlorophyll content in cotyledons, and fresh biomass weight. Macroalgal extracts also influenced epicotyl length. Algal-derived bioproducts had no phytotoxic effect on radish.

According to the statistical analysis, macroalga *C. glomerata* extracts at different concentrations significantly influenced radish growth. The obtained results are in agreement with the results obtained on radish by Kasim et al. [11] with the extract from *Codium taylorii* and *Pterocladia capillacea*; Michalak et al. [13] with *Cladophora glomerata*, *Ulva flexuosa*, *Ulva clathrata,* and *Polysiphonia fucoides*; Mahmoud et al. [3] with *Sargassum vulgare;* and Ahmed et al. [12] with dry biomass of *Ulva fasciata* and *Sargassum lacerifolium*. According to Mahmoud et al. [3], these results could be connected to the presence of higher contents of phenolic compounds and antioxidants in algal extract which might affect the metabolic processes of the tested plants. The improved seedling growth can also be attributed to the content of macro- and micronutrients, as well as amino acids, vitamins, and polysaccharides in algal extracts [11]. In the present study, the applied *C. glomerata* extract contains primary macronutrients such as K and P; secondary macronutrients such as Mg, S, and Ca; and micronutrients such as Mn, Fe, Cu, Zn, and polyphenols and exhibits antioxidant activity, as determined by DPPH analysis.

According to the data collected in Table 2 and presented in Figure 4a–e, it can be seen that root length increased after the treatment with 20–80% extracts, but decreased after the treatment with E100 as compared with the control group. The highest value (7.2 cm) was obtained for E20 and the lowest (5.3 cm) for E100. The difference between those two values was statistically significant. Mahmoud et al. [3] (for seed soaked in 3 mL/L *Sarga-ssum vulgare* extract for 12 h) and Ahmed et al. [12] (for dry biomass of *Ulva fasciata* and *Sargassum lacerifolium*) also proved positive impacts of macroalgae on radish root size. This effect can be attributed to the presence of active compounds in the extracts, such as phytohormones, polyphenols, and their antioxidant activities [3,11]. A vigorous root system can enhance water and nutrient absorption by plant roots from the soil [3].

Hypocotyl length increased for all concentrations of extracts as compared with the control. The highest value (3.3 cm) was obtained for both E60 and E80 and the lowest (2.1 cm) for distilled water used in the control group. For this parameter, statistically significant differences were observed between all macroalgal extract concentrations and the control group. Moreover, the difference between hypocotyl length for E20 and other extracts, except E40, was also statistically significant. Epicotyl length for all extracts was significantly higher than that for the control. The highest value (1.7 cm) was obtained for E60 and the lowest value (1.0 cm) was obtained for distilled water. Epicotyl length for E60 was also significantly higher than that for E20. The highest value of whole plant length (5.2 cm) was obtained for E60 and was significantly higher than that for E20 and distilled water. The lowest value (3.0 cm) was obtained for distilled water and was significantly smaller than for all extracts. Statistically significant differences were also observed between E20 and the rest of the concentrations, except E40. Studies done by Kasim et al. [11] (for 10 g/L *Codium taylorii* and *Pterocladia capillacea* extracts) and Ahmed et al. [12] (for dry biomass of *Ulva fasciata* and *Sargassum lacerifolium*) also showed positive impacts of macroalgae on radish shoot size.

Chlorophyll content in radish cotyledons increased for E40, E80, and E100 and decreased for the remaining concentrations as compared with the control group. The SPAD index was the highest for E100 (54.9) and the lowest for E20 (48.9). The difference between these two values was statistically significant. These results are in accordance with those of Kasim et al. [11] (10 g/L *Codium taylorii* and *Pterocladia capillacea* extracts), Mahmoud et al. [3] (seed soaked in 3 mL/L *Sargassum vulgare* extract for 12 h), and Ahmed et al. [12] (dry biomass of *Ulva fasciata* and *Sargassum lacerifolium*) who stated that radish treatment with macroalgae improved chlorophyll *a* and chlorophyll *b* content in leaves. The improvement in the pigment biosynthesis can be attributed to the content of phytohormones, such as cytokinin in [3], as well as high concentrations of N and Mg (structural components of chlorophyll) in algal extracts [11].

In Table 2 and Figure 4f, it can be seen that all extracts significantly increased the weight of fresh biomass. The highest value (2.44 g) was obtained for E60 and was also significantly higher than the values for E20 and distilled water. The lowest value (1.06 g) was obtained for distilled water and was significantly smaller than the weights for all of the extract concentrations. Similar results of positive impacts of macroalgae on radish bio-mass were reported by Michalak et al. [13] (*Cladophora glomerata*, *Ulva flexuosa*, *Ulva clathrata,* and *Polysiphonia fucoides* compost and extract from algae compost), Mahmoud et al. [3] (seed soaked in 3 mL/L *Sargassum vulgare* extract for 12 h), and Ahmed et al. [12] (dry biomass of *Ulva fasciata* and *Sargassum lacerifolium*).

Generally, it can be concluded that the most suitable concentration of *C. glomerata* extract for improved radish growth is 60%, because, for this concentration, the highest length of hypocotyl, epicotyl, and whole plant, as well as the weight of fresh biomass were obtained; only root length and chlorophyll content in cotyledons were lower than those for other concentrations, but the differences were not statistically significant for *p* < 0.05.

The positive influence of *C. glomerata* extract on radish seed germination and plant growth can be caused by biologically active compounds that are present in that algal biomass (carbohydrates, minerals, and proteins) and derived extract. Moreover, *C. glomerata* is a rich source of macroelements such as Mg, K, and Na, as well as microelement, i.e., Fe [7] (present work). Michalak et al. [13] studied the effect of simultaneous use of a mixture of four macroalgae (*Cladophora glomerata*, *Ulva flexuosa*, *Ulva clathrata,* and *Polysiphonia fucoides*), in the form of algal compost and compost extract on radish seed germination. The results showed that both compost and extract did not cause phytotoxic effects on radish and increased plant growth. Additionally, the biomass of plants treated with algal compost and compost extract was biofortified in micro- and macroelements (in particular B, Fe, Cu, Zn, Ca, K, and S). The same was observed by Mahmoud et al. [3], i.e., the application of extract from *Sargassum vulgare* enhanced growth and nutritional quality of red radish plants, i.e., the content of total phenolics, flavonoids, and anthocyanins in leaves and roots, as well as mineral content. Considering the stimulatory effects on plant growth and chlorophyll content, green macroalga extract of *C. glomerata* can be used as a natural plant growth stimulant in organic sustainable agriculture.

#### 2.3.2. Stimulation of Radish Growth by ZnO NPs

Some researchers have studied the impact of different types of NPs on radish, for example, silver NPs [56] and cerium oxide NPs [2]. However, there are not many studies about the effect of ZnO NPs on radish growth, development, and composition. Singh and Kumar [57], in their research, examined the toxic effect of copper oxide and zinc oxide NPs in high concentrations (from 0.1 to 1000 mg/L) on plants. The results showed that 100 and 1000 mg/L had deleterious effect on root length, shoot length, and fresh weight. Mahmoud et al. [58] investigated the simultaneous influence of ZnO NPs (at 60 mg/L) and FeO NPs (at 50 mg/L) on red radish plants. According to their research, the combined application of these two types of NPs could improve the morphological and physiological traits, as well as the nutritional quality of the plants.

In the germination tests, the effects of zinc oxide nanoparticles were compared with the control group (water), and also with the same concentrations of zinc salt, which was used to produce these nanoparticles. It is believed that nanoparticles can influence better physicochemical properties and biological activities of plants as compared with their bulk parent materials due to their size, but special attention should be paid to their mobility; reactivity; potential risks in agricultural soil; and toxicity to plants, animals, and human health through the pathways of the food chain [42].

**Figure 5:**
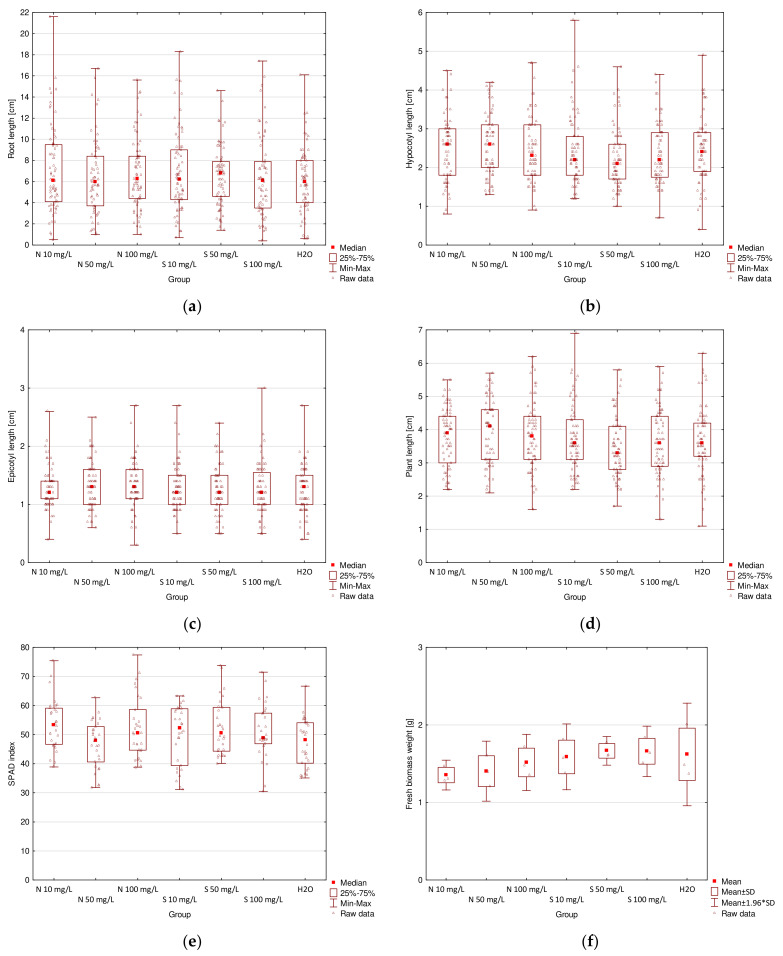
Box plots for results of germination tests with the use of various concentrations of ZnO NPs and ZnSO_4_: (**a**) root length; (**b**) hypocotyl length; (**c**) epicotyl length; (**d**) whole plant length; (**e**) chlorophyll content; (**f**) fresh biomass weight.

The summary of results for plant length, chlorophyll content, and fresh biomass weight measurements is presented in Table 3. A comparison of all the results is presented in box plots in Figure 5.

Although the differences in seedlings growth parameters and chlorophyll content in cotyledons can be observed for the treatment of radish with ZnO NPs, according to the statistical analysis with the Tukey multiple comparison test (for normal distribution and the homogeneity of variances) or the Kruskal–Wallis test (for lack of the normal distribution or the homogeneity of variances), there were no statistically significant differences for any of the examined parameters.

On the basis of the data collected in Table 3 and presented in Figure 5a–e, it can be stated that the highest root length was obtained for 50 mg/L of ZnSO_4_ (6.8 cm) and the lowest root length for both 50 mg/L of ZnO NPs and distilled water (6.0 cm). Generally, root length was the same or slightly higher for all concentrations of ZnO NPs as compared with the control group (water). The comparison of the results for ZnO NPs and for ZnSO_4_, shows that for 10 and 50 mg/L better results were obtained for groups treated with inorganic salt, but better results for 100 mg/L for ZnO NP treatment. In contrast, Singh and Kumar [57] reported a significant reduction in root length of radish for 100 mg/L ZnO NP treatment and lack of a statistically significant effect for 10 mg/L ZnO NP treatment. However, Itroutwar et al. [54] reported a significant improvement in rice (*Oryza sativa*) root length for 10 mg/L ZnO NP treatment. According to Sohail et al. [45], treatment of canola (*Brassica napus*) with 5–25 mg/L of ZnO NPs increased root length significantly.

Hypocotyl length was the highest for both 10 and 50 mg/L of ZnO NPs (2.6 cm) and the lowest for 50 mg/L of ZnSO_4_ (2.1 cm). Generally, hypocotyl length for seedlings treated with NPs was higher than that for seedlings treated with the respective concentrations of the salt. Lower concentrations (10 and 50 mg/L) of NPs resulted in an increase in hypocotyl length as compared with the control, but a higher concentration (100 mg/L) resulted in a slight decrease in hypocotyl length. Epicotyl length had the same values for 50 and 100 mg/L ZnO NPs and distilled water (1.3 cm) and was slightly lower for the rest of the groups (1.2 cm). Whole plant length value increased for all concentrations of ZnO NPs as compared with the control and stayed the same (for 10 and 100 mg/L) or decreased (for 50 mg/L) for ZnSO_4_. The highest value was obtained for 50 mg/L NPs (4.1 cm) and the lowest value was obtained for 50 mg/L salt (3.3 cm). Similarly, Singh and Kumar [51] also did not report statistically significant differences in shoot (hypocotyl) length of radish for 10 and 100 mg/L ZnO NPs. However, according to Sohail et al. [45], treatment of canola (*Brassica napus*) with 5–25 mg/L ZnO NPs and according to Itroutwar et al. [54] treatment of rice (*O. sativa*) with 10 mg/L ZnO NPs increased its shoot length significantly. Rizwan et al. [59] also reported an increase in plant height after treatment with ZnO NPs on wheat (*Triticum aestivum*) (concentrations 25–100 mg/L).

Chlorophyll content (expressed as the SPAD index) in radish cotyledons was the highest for 10 mg/L of ZnO NPs (53.4) and the lowest for 50 mg/L ZnO NPs (48.1). Treatments with both 10 and 100 mg/L NPs resulted in higher chlorophyll content as compared with the control and respective concentrations of the salt. However, for 50 mg/L ZnO NPs, it was slightly lower than that for the control and 50 mg/L of ZnSO_4_. In contrast, Venkatachalam et al. [34] reported an increase in the level of chlorophyll a and b in cotton (*Gossypium hirsutum*) for 25–200 mg/L ZnO NP treatment.

According to Table 3 and Figure 5f, it can be concluded that fresh biomass weight increased with an increase in the concentration of ZnO NPs. However, it was lower than that for all ZnSO_4_ concentrations and for distilled water. The highest value was obtained for 50 mg/L of the salt (1.67 g) and the lowest value was obtained for 10 mg/L NPs (1.35 g). A study conducted by Singh and Kumar [57] also confirmed that the radish treatment with 10 and 100 mg/L ZnO NPs reduced fresh biomass weight. However, Rossi et al. [60] reported a positive influence on fresh biomass weight of coffee plant (*Coffea arabica*) treated with 10 mg/L of ZnO NPs and Venkatachalam et al. [36] reported a positive influence on cotton (*G. hirsutum*) treated with nanoparticles with concentrations varying from 25 to 200 mg/L.

According to the presented results, it can be stated that mostly smaller concentrations (10 or 50 mg/L) gave better results than a higher concentration (100 mg/L). However, the effects of the radish treatment with ZnO NPs were not different at a statistically significant level from the effects caused by ZnSO_4_ treatment or the control group. This suggests that the applied concentrations of ZnO NPs were too high for radish. According to the literature, although a concentration of 10 mg/L can improve plant growth parameters significantly for rice (*O. sativa*) [54] or coffee plant (*C. arabica*) [60], smaller concentrations (about 0.1–1.0 mg/L) should show better results for radish (*R. sativus*) [57].

ZnO NPs have been reported to have both positive and negative impacts on seed germination and plant growth, depending on the concentration used, plant tested, and other conditions. According to Rizwan et al. [59], seed priming with mineral NPs may change the nutrient contents in seeds which affects plant growth, yield, and quality. Metal containing nanoparticles, due to their size and large surface area-to-volume size ratio, after foliar or root application are easily transported in the plant system [54]. It has been confirmed that ZnO NPs could improve germination parameters [45,59,60,61,62,63,64] and chlorophyll content [36,65,66]. It is hard to indicate which concentrations are the most effective for improving plant growth parameters, as the most effective concentration varies among the many parameters, especially tested plant species and method of application of NPs.

### 2.4. Adsorption Kinetics and Equilibrium of Cr(III) Ions

**Figure 6:**
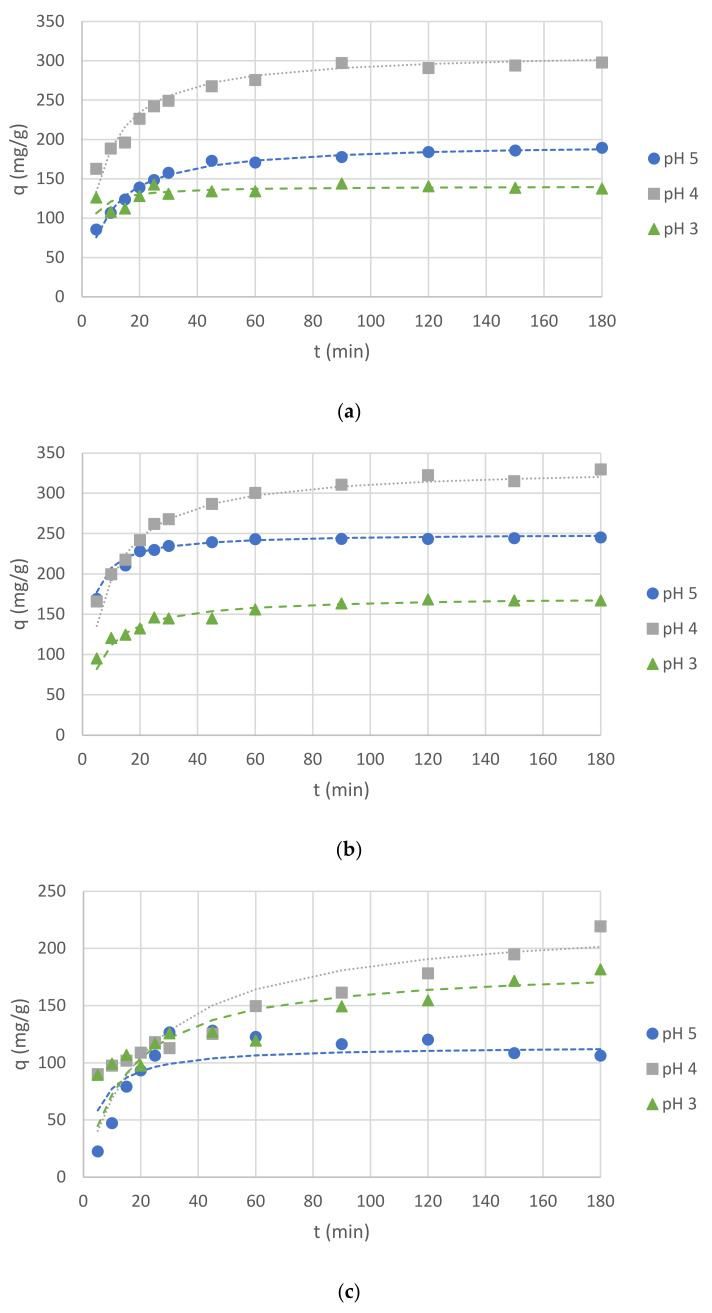
Effects of pH on Cr(III) ion sorption capacity (*C*_0_, 300 mg/L and *C*_S_, 1 g/L) for: (**a**) *C. glo-merata* biomass; (**b**) *C. glomerata* post-extraction residue; (**c**) ZnO NPs.

**Figure 7:**
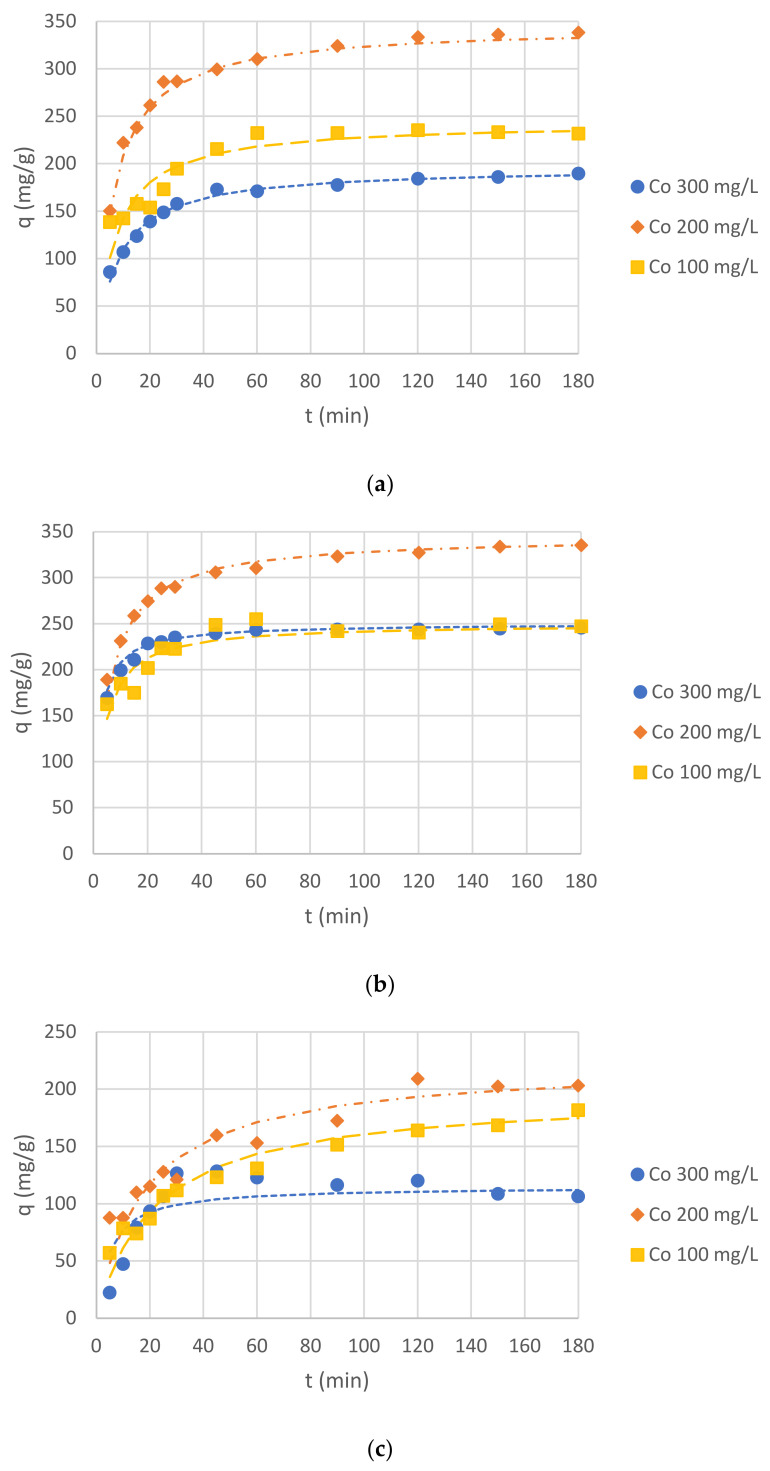
Effects of *C*_0_ on Cr(III) ion sorption capacity (pH of 5 and *C*_S_, 1 g/L) for: (**a**) *C. glomerata* biomass; (**b**) *C. glomerata* post-extraction residue; (**c**) ZnO NPs.

**Figure 8:**
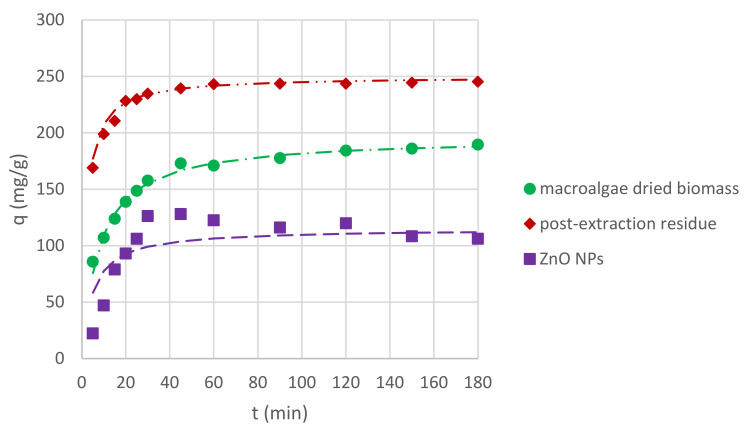
Effects of sorbent type on Cr(III) ion sorption capacity (pH of 5; *C_S_*, 1 g/L; and *C*_0_, 300 mg/L).

In order to analyze the results of Cr(III) ion sorption by *C. glomerata* dried biomass, *C. glomerata* post-extraction dried residue, and ZnO NPs, the pseudo-first-order and the pseudo-second-order kinetic models were applied. The pseudo-second-order kinetic model turned out to be more suitable (the values of R^2^, i.e., the correlation coefficients for the pseudo-first-order kinetic model, were lower than 0.5 in some cases); therefore, only the results obtained for this model are presented. The obtained values of q_eq_ (mg/g), k_2_ (g/(mg·min)), and R^2^ for the pseudo-second-order kinetic model for different pH values are presented in Table 4 and for different initial concentrations of Cr(III) ions in the solution in Table 5. The effect of the initial pH on the sorption of Cr(III) ions for all sorbents is shown in Figure 6, while the effect of the initial concentration of Cr(III) ions is shown in Figure 7. For the initial pH of 5 and the concentration of Cr(III) ions of 300 mg/L, the effect of different sorbent types on the sorption capacity was also compared (Figure 8).

The presented study results proved that all bioproducts, i.e., *Cladophora glomerata* dried biomass, *Cladophora glomerata* post-extraction residue, and biosynthesized ZnO NPs can be used as sorbents of Cr(III) ions from aqueous solutions. On the basis of Figure 6 and Figure 7 it can be observed that for all types of sorbents and for all initial concentrations of Cr(III) ions and the initial pH values, adsorption capacities increased along with contact time. At first, it increased rapidly, up to 60 min, and then it began to increase slower until it reached equilibrium sorption capacity. On the basis of sorption capacity values at equilibrium (Table 4 and Table 5), it can be seen that the best results were reached for both *C. glomerata* dried biomass and *C. glomerata* post-extraction residue for an initial pH of 5 and an initial concentration of Cr(III) ions of 200 mg/L. The value of q_eq_ was 345 mg/g for both sorbents.

#### 2.4.1. Effect of Initial pH on Sorption Capacity

The sorption capacity was examined for different values of initial pH, which were chosen based on the analysis of the literature. For Cr(III) ions, when the pH value was above 5, the solution became cloudy. The precipitate indicated the occurrence of chromium (III) hydroxide [67]. Therefore, the highest pH value chosen for this experiment was 5, which is the value most often used for Cr(III) ion biosorption by macroalgal biomass in the literature [16,17,18,19]. For comparison, lower pH values (4 and 3) were also investigated. Hamdy [68] showed that for some macroalgae types, a lower pH was more effective for Cr(III) ion biosorption. When analyzing *q*_eq_ values for different initial pH values (Table 4), it can be stated that, for all three sorbents (biosorbents), the highest values were obtained for an initial pH value of 4, which means that this pH level was the most suitable for carrying out the sorption of Cr(III) ions. The best biosorption capacity for all examined conditions was observed by *C. glomerata* post-extraction residue.

#### 2.4.2. Effect of Initial Cr(III) Ion Concentrations on Sorption Capacity

The second factor influencing the sorption process, which was examined in this study, was the initial concentration of Cr(III) ions in the solution. It is known that the sorption process depends on the initial metal ion concentration [20], but it cannot be claimed if higher or lower concentrations are more suitable, as the sorption process depends on other factors (e.g., pH, temperature, biosorbent dose, or adsorption time) which should be investigated for all types of sorbents (biosorbents) [48]. In our study, three concentrations were examined: 100, 200, and 300 mg/L. The highest q_eq_ values were obtained for 200 mg/L (max. 345 mg/g) and the lowest q_eq_ values were obtained for 300 mg/L (max. 208 mg/g) for all sorbents (biosorbents). Probably, the concentration of 300 mg/L of Cr(III) ions was too high for the amount of sorbents (biosorbents) used (i.e., 1 g/L) to effectively adsorb the metal ions.

#### 2.4.3. Effect of Adsorbent Type

The types of adsorbents used in this study are compared in Figure 6. The conditions chosen for the comparison were as follows: initial pH of 5, initial Cr(III) ion concentration of 300 mg/L, and *C_S_* of 1 g/L. The highest sorption capacity was reached for post-extraction residue of *C. glomerata* and the lowest sorption capacity was reached for ZnO NPs. By comparing the *q*_eq_ values for other conditions collected in Table 4 and Table 5, it can be seen that for an initial pH of 4 and a concentration of 300 mg/L and an initial pH of 5 and a concentration of 100 mg/L, the dependence is the same. However, for an initial pH of 5 and a concentration of 200 mg/L, the value of the sorption capacity at equilibrium is the same for both *C. glomerata* dried biomass and *C. glomerata* post-extraction residue and lower for ZnO NPs. For an initial pH of 3 and a concentration of 100 mg/L, the sorption capacity is the highest for ZnO NPs and the lowest for *C. glomerata* dried biomass.

The obtained values of *q*_eq_ are quite high as compared with those in the literature data. Usually, the biosorption capacity of Cr(III) ions for macroalgae is below 100 mg/g [16,17,18,69]. Results higher than 100 mg/g were obtained by Guarín-Romero et al. [19] (102 mg/g for brown macroalga *Durvillaea antarctica*) and Ibrahim [17] (105 mg/g for red macroalga *Galaxaura oblongata*). However, it is difficult to compare the results, since most of the researchers used higher amounts of biosorbent (up to 25 g/L by Tamilselvan et al. [18]) and lower initial Cr(III) ion concentrations, i.e., 5 mg/L by Ibrahim [17] and 52 mg/L by Murphy et al. [69]. The study with the most similar conditions was carried out by Guarín-Romero et al. [19] who used a pH from 2.0 to 5.5, biosorbent dose of 1 g/L, and an initial Cr(III) concentration of 7.5–300 mg/L and obtained a maximum biosorption capa-city (*q*_max_) of 100 mg/g.

There are still only a few studies on biosorption of heavy metals by post-extraction residue obtained from the extraction of active compounds from macroalgae. Cardoso et al. [35] investigated the ability of the residue from alginate extraction of *Sargassum filipendula* to adsorb Cr(III) ions, but the *q*_eq_ value was very low (5.72 mg/g for a pH of 3.5, *C*_S_ of 2.0 g/L, and *C*_0_ of 52 mg/L). Better results were obtained by Li et al. [32], who studied the ability of residue from polysaccharides extraction from brown macroalga *Sargassum carpophyllum* and green macroalga *Caulerpa lentillifera* to adsorb Cd(II), Cu(II), Mn(II), and Pb(II) ions. The obtained values of *q*_eq_ were up to 109 mg/g (pH of 2–6, *C*_S_ of 0.8 g/L, and *C*_0_ of 1–10 mg/L). In addition, Mohamed et al. [34] evaluated the residue of *Padina gymnospora* waste after extraction of its active components as a promising low-cost adsorbent (*q*_eq_ 31.5 mg/g for Cr(III) and 96.5 mg/g for Cd(II), pH of 6.2, 25 °C, and *C*_0_ of 100 mg/L).

#### 2.4.4. Sorption Equilibrium of Cr(III) Ions

**Figure 9:**
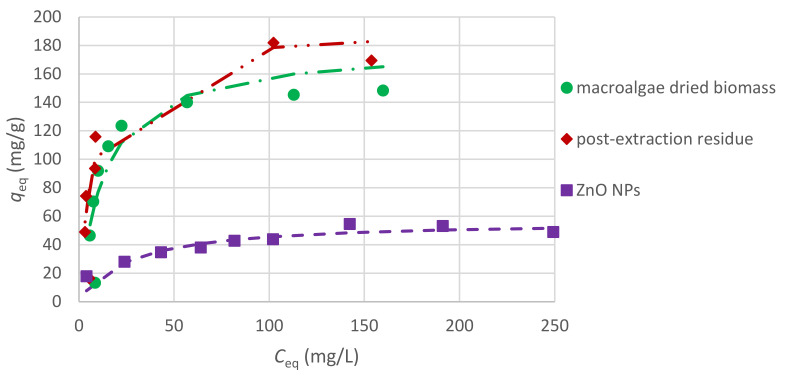
Adsorption isotherms for different sorbents (*C*_0_ of 25–300 mg/L and *C*_S_ of 10 g/L, and pH of 5).

The results obtained in the experiments on sorption equilibrium were fitted to the Langmuir adsorption isotherm, which assumes monolayer adsorption on the surface of the sorbent. According to the Langmuir model (Equation (6)), the values of the maximum sorption capacity are: *q*_max_ for *C. glomerata* biomass was 169 mg/g and constant *b* was 0.0669 L/mg; for *C. glomerata* post-extraction residue, *q*_max_ was 192 mg/g and *b* was 0.142 L/mg; and for ZnO NPs, *q*_max_ was equal to 57 mg/g and *b* was 0.0389 L/mg. The adsorption isotherms for the examined sorbents (biosorbents) are presented in Figure 9. The tested biosorbents showed comparable sorption properties, which were much higher than those for zinc oxide nanoparticles.

It is difficult to compare the maximum sorption capacity of different sorbents to adsorb Cr(III) ions, because sorption has been performed in different experimental conditions. For example, Ibrahim obtained a *q*_max_ of 105 mg/g for *C*_S_ of 10 g/L and pH of 5 for *Galaxaura oblongata* [17] and Tamilselvan et al. obtained a *q*_max_ of 79.6 mg/g for *C*_S_ of 25 g/L, pH of 5, and *C*_0_ of 50–200 mg/L using *Sargassum wightii* as a biosorbent [18]. Seaweeds exhibit good biosorption properties to adsorb heavy metal ions and are considered to be a promising, cheap, efficient, and biodegradable sorbent for treating industrial effluents [17,18].

## 3. Materials and Methods

### 3.1. Chemicals

Zinc sulphate heptahydrate, sodium hydroxide micropills, and disodium versenate dihydrate (EDTA) were purchased from Avantor Performance Materials (Gliwice, Poland). Chromium (III) nitrate nonahydrate was purchased from Honeywell Fluka™ (Charlotte, NC, USA) and hydrochloric acid 35–38% (*m*/*m*) was purchased from Chempur^®^ (Piekary Śląskie, Poland). EMSURE ACS grade concentrated HNO_3_ (65%, *m*/*m*) was obtained from Merck (KGaA, Darmstadt, Germany) and used for sample digestion. A commercially available Merck Certipur (Merck, KGaA, Darmstadt, Germany) multielemental stock (100 mg/L) ICP standard solution (no. IV) and single ICP stocks (1000 mg/L) of As, Hg, P, S, and Se (Merck, KGaA, Darmstadt, Germany) were used for preparing standard solutions for calibration of the ICP-OES instrument. Deionized water (18.3 MΏ cm) from an EASYpure RF water purification system (Barnstead/Thermolyne Corporation, Dubuque, IA, USA), model D7033, was used throughout. Gallic acid monohydrate, Trolox, and DPPH were purchased from Sigma-Aldrich (Saint Louis, MI, USA). Folin–Ciocalteu’s phenol reagent was purchased from Merc KGaA (Darmstadt, Germany).

### 3.2. Freshwater Macroalgae Biomass

The biomass of *Cladophora glomerata* (freshwater macroalgae) was collected from the lake’s surface in the village Tomaszówek (Łódź Province, Poland) in October 2016. The collected macroalgae were identified based on morphological characteristics according to the taxonomic literature for this area [70]. Then, the biomass was air-dried and milled using a grinding mill (Retsch GM300, Haan, Germany). During the sieve analysis (Retsch, Haan, Germany), the biomass with particle size lower than 400 μm was chosen.

### 3.3. Production of the Macroalgal Extract

Macroalgal extract was produced according to the methodology described by Michalak et al. [49]. Dry *C. glomerata* biomass (4 g) was suspended in an aqueous solution (80 mL). The mixture was subjected to the ultrasound homogenizer for 30 min working at parameters: 50 W, ultrasonic frequency 30 kHz, amplitude 100% (UP 50H, Hielscher Ultrasonics, Teltow, Germany). Then, the mixture was centrifuged for 10 min at a speed rate of 4000 rpm. The obtained supernatant was treated as 100% (E100) macroalgal extract. The pH of the extract was measured in 4 replications with a pH meter (Mettler Toledo SevenMulti (Mettler-Toledo, Warsaw, Poland)). This concentration was used to prepare 20% (E20), 40% (E40), 60% (E60), and 80% (E80) extracts. Post-extraction residue was at first air-dried, then, milled using a grinding mill, and collected for further use as a biosorbent.

### 3.4. Synthesis of Zinc Oxide Nanoparticles

The zinc oxide nanoparticles were produced according to the methodology described by [53] with minor changes. Then, 100 mL of 100 mM zinc sulphate heptahydrate solution was prepared and kept in an incubator with a shaker set at 60 °C and 250 rpm. 15 mL of *C. glomerata* 100% extract was added dropwise. Next, the level of pH was measured (6.07 ± 0.28) with a pH meter and adjusted to 12 by the addition of 1 M solution of NaOH. A white cloudy appearance marked the formation of ZnO NPs. The solution was left for two hours in the same conditions in the incubator with a shaker. Then, it was incubated for twenty hours at room temperature. After this time, the solution was centrifuged for 10 min at 4000 rpm. The white pellet was collected and air-dried at 80 °C. Then, it was ground in the mortar to obtain white powder. The synthesis of NPs was made in 4 replications.

### 3.5. Germination Tests

In the present study, red radish seeds (*Raphanus sativus*), cv. Pharaoh from WerbAna Sp. z o.o. (Warsaw, Poland) were used. Germination tests were conducted on a filter paper to investigate the phytotoxicity of the tested algal products. Tests were carried out on Petri dishes in 3 replications, 25 seeds per replication. Each replication in every group was watered with 3 mL of required solution or distilled water.

First, the germination test consisted of 6 experimental groups: 5 groups treated with different concentrations of macroalgal extract (20, 40, 60, 80, and 100%) and a control group watered with distilled water. The average temperature during this experiment was 22.1 ± 1.2 °C and the average air humidity was 44 ± 5%.

The second germination test consisted of 7 groups: 3 groups treated with different concentrations of ZnO NP solutions (10, 50, and 100 mg/L), 3 groups treated with the same concentrations of ZnSO_4_ solutions, and a control group watered with distilled water. The concentrations were chosen based on the literature analysis [54]. During preparation of NPs solutions for germination tests, the mixture of NPs and water was subjected to an ultrasound homogenizer for 10 min working at parameters: 50 W, ultrasonic frequency 30 kHz, and amplitude 100%. The average temperature during this experiment was 20.8 ± 0.6 °C and the average air humidity was 44 ± 2%.

The germination tests both lasted 11 days. After this time, the seedling parameters root length, hypocotyl length (the stem of a germinating seedling is found below the cotyledons and above the root), epicotyl length (the embryonic shoot above the cotyledons), whole plant length, fresh biomass weight, and chlorophyll content in cotyledons were measured.

### 3.6. Adsorption of Cr(III) Ions

#### 3.6.1. Adsorption Kinetics of Cr(III) Ions

Three biosorbents were tested in this study: *C. glomerata* biomass, post-extraction residue of *C. glomerata*, and ZnO NPs synthesized with the use of *C. glomerata* extract. The experimental conditions were as follows: (a) C_S_ of 1 g/L, C_0_ of 300 mg/L, pH of 3, 4, and 5 and (b) C_S_ of 1 g/L, pH of 5, C_0_ of 100, 200, and 300 mg/L, where C_0_ is the initial concentration of Cr(III) ions in the solution. The selection of experimental conditions was based on previous research [71]. The pH value was measured and adjusted to the appropriate value by the addition of NaOH or HCl. To 200 mL of each solution, 0.2 g of biosorbent (*Cladophora glomerata* dried biomass or dried post-extraction residue of *C. glomerata*) or sorbent (zinc oxide nanoparticles) was added. The mixtures were stirred for 3 h at 200 rpm and room temperature. After 5, 10, 15, 20, 25, 30, 45, 60, 90, 120, 150, and 180 min, respectively, 7 mL of each mixture was taken and filtered through a filter paper. In addition, the pH value in mixtures after 3 h of stirring was measured.

The sorption capacity, *q* (mg/g), was calculated using the equation:

(1) $$q=\frac{C_{0}-C_{t}}{C_{s}}\;$$

where *C*_0_ (mg/L) is the initial concentration of Cr(III) ions in the solution, *C*_t_ (mg/L) is the concentration of Cr(III) ions in given time t, and *C_s_* (g/L) is the concentration of sorbent (or biosorbent) in the solution.

The determination of kinetics is very important for the design of adsorption systems. Adsorption kinetics describes the relations between the amount of adsorbates adsorbed on the adsorbent (sorption capacity) and the contact time. The most often used models for the description of adsorption kinetics are the pseudo-first-order kinetic model and the pseudo-second-order kinetic model [34,72,73].

The pseudo-first-order kinetic model of Lagergren is expressed as follows:

(2) $$\frac{dq_{t}}{dt}=k_{1}\left(q_{\mathrm{eq}}-q_{t}\right)\;$$

and its integral form as follows:

(3) $$\log\left(q_{\mathrm{eq}}-q_{t}\right)=logq_{\mathrm{eq}}-\frac{k_{1}}{2.303}t$$

where *q*_eq_ (mg/g) is the equilibrium adsorption capacity of the adsorbent, *q*_t_ (mg/g) is the adsorption capacity in given time t, and *k*_1_ is the rate constant of the pseudo-first-order adsorption model (1/min).

The pseudo-second-order kinetic model is expressed as follows:

(4) $$\frac{dq_{t}}{dt}=k_{2}{\left(q_{\mathrm{eq}}-q_{t}\right)}^{2}$$

and its integral form as follows:

(5) $$\frac{t}{q_{t}}\;=\;\frac{1}{k_{2}q_{\mathrm{eq}}{}^{2}}+\frac{1}{q_{\mathrm{eq}}}t$$

where *k*_2_ is the rate constant of the pseudo-second-order adsorption model (g/(mg∙min)) [73].

#### 3.6.2. Sorption Equilibrium of Cr(III) Ions

Nine solutions of Cr(III) ions at concentartions 25, 50, 75, 100, 125, 150, 200, 250, and 300 mg/L were prepared. The pH was adjusted to 4 (the value was chosen after analyzing the results of adsorption kinetics). To 40 mL of each solution, 0.04 g of biosorbents (*Cladophora glomerata* biomass or post-extraction residue of *C. glomerata*) or sorbent (zinc oxide nanoparticles) was added. The mixtures were stirred at 100 rpm, for 3 h, at room temperature. After 3 h of stirring, 10 mL of each mixture was taken and filtered through a filter paper.

The values of the maximum sorption capacity for the tested sorbents were calculated from the Langumir model (6) after its linearization (7):

(6) $$q_{\mathrm{eq}}\;=\;q_{\max}\cdot \frac{b\cdot C_{\mathrm{eq}}}{1+b\cdot C_{\mathrm{eq}}}$$

(7) $$\frac{C_{\mathrm{eq}}}{q_{\mathrm{eq}}}\;=\;\frac{1}{bq_{\max}}+\frac{C_{\mathrm{eq}}}{q_{\max}}$$

where *C*_eq_ is the equilibrium concentration of the Cr(III) ions (mg/L) in the solution, *b* is the adsorption equilibrium constant (L/mg), and *q*_max_ is the maximum sorption capacity (mg/g) [34,73].

### 3.7. Analytical Techniques

#### 3.7.1. Multielemental Analysis

The multielemental analyses of the raw algal biomass, 100% extract, post-extraction residue, and biosynthesized ZnO NPs were performed using the ICP-OES technique (inductively coupled plasma-optical emission spectrometry). This analysis was preceded by the microwave-assisted closed-vessel digestion, using a Multiwave PRO microwave reaction system (Anton Paar GmbH, Wundschuh, Austria), equipped with a 24HVT50 rotor. Accordingly, approximately 0.25 g of samples or 5.0 mL of extracts were placed into PTFE vessels and 5.0 mL of concentrated HNO_3_ was added. Then, the vessels were closed, inserted into the rotor and digested in a microwave reaction system employing a 6-step microwave-assisted heating program with a maximum temperature of 190 °C for 60 min. The resulting sample remnants were allowed to cool to room temperature, quantitatively transferred into 30 mL polypropylene containers (Equimed, Kraków, Poland), diluted with deionized water to 25 g, and kept at 4 °C until measurement. All samples were analyzed in triplicate (N = 3). With each set of digested samples, blanks were simultaneously prepared and considered in the final results. To avoid differences in density, sample solutions were prepared by weight. The spectrometric measurements were made using an Agilent bench-top simultaneous optical emission Ar-ICP spectrometer (model 720) with the torch in the axial alignment. External 6-point calibration curves, spanning the 0–5.0 mg/L concentration range, were used to quantify the contents of the studied elements.

#### 3.7.2. Determination of the Total Value of the Polyphenols in Algal Extract

The total content of phenolic compounds in the extract was determined according to the ISO 14502-1 standard using Folin–Ciocalteu reagent [74]. Gallic acid was used to prepare the standard curve. The absorbance was measured at a wavelength of 765 nm. The concentration of phenolic compounds in extract was expressed as mg/L of gallic acid equivalents (GAE). The analyses were performed in triplicate.

#### 3.7.3. Determination of Antioxidant Activity of Algal Extract by the DPPH Method

The antioxidant activity of extract was determined by the modified method described by Brand-Wiliams et al. with the use of synthetic radical DPPH [75]. Trolox was used to prepare the standard curve. The absorbance was measured at a wavelength of 515 nm. The antioxidant activity was expressed as Trolox equivalent. The analyses were performed in triplicate.

#### 3.7.4. Chlorophyll Measurement

For the measurement of the chlorophyll content in radish cotyledons, a handheld SPAD chlorophyll meter (Konica Minolta, Tokyo, Japan) was used, which provided a relative index of chlorophyll content in seedlings. In each experimental group and in each repetition, 10 measurements were made from randomly selected seedling cotyledons.

#### 3.7.5. Characteristics of Nanoparticles

The UV-Vis spectrophotometric analysis of synthesized ZnO nanoparticles was performed with a GENESYS™ 10S UV-Vis Spectrophotometer (ThermoFisher Scientific, Waltham, MA, USA). The solution was diluted 5 times before measurements.

The surface of the samples was examined using a scanning electron microscopy (Quanta 250, FEI). The surface topography was evaluated using an SE detector. The samples were sputtered with gold with a thickness of about 10 nm (LEICA EM ACE200). This was due to the improvement in the discharge of electric charge from the surface of the samples and to ensure proper surface stability of ZnO samples during microscopic observations.

#### 3.7.6. Determination of Cr(III) Ions in the Solution

In order to determine the concentration of Cr(III) ions in the solutions before and after sorption, 4 mL of each filtrate was added to 0.095 g of EDTA and heated (95 °C) for 10 min. The EDTA was added to create Cr(III)–EDTA violet complex formations, which enabled us to measure the chromium concentration. The absorbance of filtrates was measured with a Spectrophotometer Biosens UV 5100 (Warsaw, Poland) at wavelength 540 nm [71,76].

### 3.8. Statistical Analysis

The measured values were elaborated statistically by Statistica version 13.0 (TIBCO Software Inc., Tulsa, OK, USA). Descriptive statistics (average and standard deviations or median and quartile range, Q25–Q75) for all experimental groups were performed. The normality of the distribution of experimental results was assessed by the Shapiro–Wilk test and the homogeneity of variances by the Brown–Forsythe test. On this basis, the statistical test, which was used to investigate the significance of differences between the tested groups, was selected. The differences between several groups were investigated with the one-way analysis of variance (ANOVA) using the Tukey multiple comparison test (for normal distribution and the homogeneity of variances) or the Kruskal–Wallis test (for lack of the normal distribution or the homogeneity of variances). Median and interquartile range (Q25–Q75) were compared instead of mean and standard deviation when there was no normal distribution for the analyzed parameters. The results were considered to be significantly different when *p* < 0.05.

## 4. Conclusions

In the present study, it was shown that the waste biomass of *Cladophora glomerata* can be valorized into products useful for sustainable agriculture and wastewater treatment. Algal extracts at five concentrations (20, 40, 60, 80, and 100%) and ZnO NPs at three concentrations (10, 50, and 100 mg/L) were used to investigate their impacts on red radish (*Raphanus sativus*) seedling growth. The use of algal extract (especially 60%) gave the most promising results, according to the measurements of root length, hypocotyl length, epicotyl length, whole plant length, and fresh biomass weight. The results obtained for NPs also showed their ability to increase plant growth parameters; however, not at a statistically significant level, which means that further studies for different concentrations are required before using them on a larger scale. *C. glomerata* (in the form of dry biomass or as a post-extraction residue) and ZnO NPs were also both successful in Cr(III) ion sorption from aqueous solution.

Future perspectives will concern (1) the detailed characteristics of the algal extract, which will enable the explanation of its mechanism of action; (2) pot experiments or field tests with algal extracts and metal containing nanoparticles in order to confirm their effectiveness, i.e., positive effect on plant growth, yield and nutritional quality; (3) an explanation of the role of nanoparticles in plants, their mode of action, and potential toxicity to plants; and (4) the sorption of heavy metal ions in a multimetal system, reflecting the composition of wastewater.

## Figures and Tables

**Table 1:** The multielemental compositions of raw algae biomass (mg/kg dry biomass), algal extract (mg/L), post-extraction residue (mg/kg), and ZnO NPs synthesized from algal extract (mg/kg).

	Mean ± SD			
Element/Wavelength (nm)	*C. glomerata* Biomass	*C. glomerata* Extract	*C. glomerata* Post-Extraction Residue	ZnO NPs
Al/396.152	263 ± 5	2.55 ± 0.01	250 ± 7	114 ± 1
As/188.980	3.66 ± 0.24	0.058 ± 0.001	2.65 ± 0.16	<0.0033
B/249.772	54.1 ± 0.4	0.136 ± 0.004	57.6 ± 1.1	18.3 ± 0.1
Ba/455.503	101 ± 1	0.204 ± 0.001	113 ± 2	2.02 ± 0.06
Ca/396.847	164,829 ± 906	198 ± 1	180,769 ± 2287	2263 ± 20
Cd/214.439	<0.00029	<0.00029	<0.00029	1.10 ± 0.01
Cr/267.716	0.379 ± 0.033	0.033 ± 0.002	1.55 ± 0.12	59.4 ± 1.2
Cu/327.395	0.714 ± 0.039	0.016 ± 0.001	8.26 ± 0.16	13.5 ± 0.4
Fe/238.204	571 ± 4	4.88 ± 0.02	566 ± 4	297 ± 7
Hg/253.652	<0.0027	<0.0027	<0.0027	<0.0027
K/766.491	22,212 ± 71	872 ± 10	8252 ± 92	522 ± 5
Mg/285.213	1973 ± 30	31.4 ± 0.1	1610 ± 18	578 ± 3
Mn/257.610	139 ± 1	1.54 ± 0.01	129 ± 2	25.4 ± 0.2
Na/589.592	518 ± 10	18.3 ± 0.1	246 ± 1	13,580 ± 39
P/213.618	1008 ± 15	21.1 ± 0.3	779 ± 9	215 ± 3
Pb/220.353	<0.0024	<0.0024	<0.0024	18.7 ± 0.3
S/181.972	16,247 ± 112	284 ± 6	13,670 ± 87	10,154 ± 117
Se/196.026	<0.0068	<0.0068	<0.0068	<0.0068
Sr/407.771	203 ± 1	0.581 ± 0.002	221 ± 4	3.73 ± 0.03
Zn/213.857	6.97 ± 0.25	0.070 ± 0.003	6.15 ± 0.08	699,621 ± 9894

**Table 2:** Biometric parameters of radish treated with different concentrations of *C. glomerata* extracts.

Parameter	Root Length (cm) *			Hypocotyl Length (cm) *			Epicotyl Length (cm) *			Whole Plant Length (cm) *			SPAD Index (-) *			Fresh Biomass Weight (g) (N = 3) **		Fresh Biomass Weight per Seedling (g)	No. of Germinated Seedlings
Group	Q25	Median	Q75	Q25	Median	Q75	Q25	Median	Q75	Q25	Median	Q75	Q25	Median	Q75	Mean	SD	Mean	-
20%	4.7	7.2 ^a^	8.6	2.0	2.5 ^bcde^	3.0	1.1	1.4 ^jk^	1.7	3.5	3.8 ^pqrs^	4.6	42.5	48.9 ^x^	53.0	1.834 ^yz^	0.242	0.076	72
40%	4.5	6.2	8.3	2.4	2.9 ^f^	3.4	1.3	1.6 ^l^	1.9	3.7	4.5 ^t^	5.1	46.2	53.1	57.0	2.146 ^a’^	0.033	0.093	69
60%	4.6	6.2	8.4	2.6	3.3 ^bg^	3.9	1.4	1.7 ^jm^	2.0	4.2	5.2 ^pu^	5.8	46.0	50.4	53.1	2.445 ^yb’^	0.186	0.100	73
80%	3.9	5.8	7.3	2.5	3.3 ^ch^	3.8	1.3	1.6 ^n^	1.9	4.0	4.8 ^qv^	5.7	49.2	54.6	60.2	2.128 ^c’^	0.241	0.093	69
100%	4.1	5.3 ^a^	6.8	2.5	3.1 ^di^	4.2	1.2	1.5 ^o^	1.9	3.6	4.9 ^rtw^	6.1	50.2	54.9 ^x^	61.0	2.007 ^d’^	0.067	0.082	73
H_2_O	3.5	5.6	7.4	1.6	2.1 ^efghi^	2.3	0.8	1.0 ^klmno^	1.2	2.6	3.0 ^suvw^	3.5	39.2	51.1	56.5	1.055 ^za’b’c’d’^	0.147	0.046	69

a, b, c, … —differences statistically significant for *p* < 0.05; * Kruskal–Wallis test (results presented as a median); ** Tukey multiple comparison test (results presented as a mean).

**Table 3:** Biometric parameters of radish treated with different concentrations of ZnO NPs and ZnSO_4_.

Parameter	Root Length (cm) *			Hypocotyl Length (cm) *			Epicotyl Length (cm) *			Whole Plant Length (cm) *			SPAD Index (-) *			Fresh Biomass Weight (g) (N = 3) **		Fresh Biomass Weight per Seedling (g)	No. of Germinated Seedlings
Group	Q25	Median	Q75	Q25	Median	Q75	Q25	Median	Q75	Q25	Median	Q75	Q25	Median	Q75	Mean	SD	Mean	-
N 10	4.1	6.1	9.5	1.8	2.6	3.0	1.1	1.2	1.4	3.0	3.9	4.4	46.7	53.4	59.1	1.354	0.099	0.059	69
N 50	3.7	6.0	8.4	2.0	2.6	3.1	1.0	1.3	1.6	3.1	4.1	4.6	40.6	48.1	52.8	1.405	0.198	0.065	65
N 100	4.4	6.3	8.4	1.8	2.3	3.1	1.1	1.3	1.6	3.1	3.8	4.4	44.7	50.6	58.6	1.516	0.185	0.066	69
S 10	4.3	6.2	9.0	1.8	2.2	2.8	1.0	1.2	1.5	3.1	3.6	4.3	39.4	52.3	58.9	1.588	0.217	0.067	71
S 50	4.6	6.8	7.9	1.7	2.1	2.6	1.0	1.2	1.5	2.8	3.3	4.1	44.3	50.7	59.4	1.666	0.094	0.068	73
S 100	3.5	6.1	8.0	1.8	2.2	2.2	1.0	1.2	1.6	2.9	3.6	4.4	46.9	48.9	57.4	1.661	0.166	0.073	68
H_2_O	4.0	6.0	8.4	1.9	2.4	2.4	1.0	1.3	1.5	3.2	3.6	4.2	40.2	48.2	54.1	1.620	0.338	0.070	69

* Kruskal–Wallis test (results presented as a median); ** Tukey multiple comparison test (results presented as a mean).

**Table 4:** Parameters of pseudo-second-order kinetic model for sorption of Cr(III) ions by the examined sorbents (biosorbents) (*C*_S_, 1 g/L and *C*_0_, 300 mg/L) in different pH.

	*C. glomerata* Biomass			*C. glomerata* Post-Extraction Residue			ZnO NPs		
Initial pH	*q*_eq_ (mg/g)	*k*_2_ (g/(mg ∙ min))	*R* ^2^	*q*_eq_ (mg/g)	*k*_2_ (g/(mg ∙ min))	*R* ^2^	*q*_eq_ (mg/g)	*k*_2_ (g/(mg ∙ min))	*R* ^2^
5	196	0.00064	0.9997	208	0.00219	0.9999	115	0.00180	0.9789
4	313	0.00048	0.9995	333	0.00041	0.9992	227	0.00019	0.9698
3	141	0.00438	0.9991	172	0.00106	0.9994	185	0.00035	0.9800

**Table 5:** Parameters of pseudo-second-order kinetic model for sorption of Cr(III) ions by the examined sorbents (biosorbents) (*C*_S_, 1 g/L and pH of 5) in different concentrations (*C*_0_) (mg/L).

	*C. glomerata* Biomass			*C. glomerata* Post-Extraction Residue			ZnO NPs		
*C*_0_ (mg/L)	*q*_eq_ (mg/g)	*k*_2_ (g/(mg ∙ min))	*R* ^2^	*q*_eq_ (mg/g)	*k*_2_ (g/(mg ∙ min))	*R* ^2^	*q*_eq_ (mg/g)	*k*_2_ (g/(mg ∙ min))	*R* ^2^
300	196.1	0.00064	0.9997	208.3	0.00219	0.9999	114.9	0.00180	0.9789
200	344.8	0.00044	0.9999	344.8	0.00056	0.9999	222.2	0.00025	0.9887
100	243.9	0.00058	0.9979	250.0	0.00114	0.9988	196.1	0.00023	0.9914

## Sample Availability

Samples of the compounds are available from the authors.

## References

[1] Zhang J., He P., Ding W., Xu X., Ullah S., Abbas T., Ai C., Li M., Cui R., Jin C. (2019). Estimating nutrient uptake requirements for radish in China based on QUEFTS model. Sci. Rep..

[2] Zhang W., Ebbs S.D., Musante C., White J.C., Gao C., Ma X. (2015). Uptake and accumulation of bulk and nanosized cerium oxide particles and ionic cerium by radish (*Raphanus sativus* L.). J. Agr. Food Chem..

[3] Mahmoud S.H., Salama D.M., El-Tanahy A.M.M., Abd El-Samad E.H. (2019). Utilization of seaweed (*Sargassum vulgare*) extract to enhance growth, yield and nutritional quality of red radish plants. Ann. Agric. Sci..

[4] Pikosz M., Messyasz B. (2016). Characteristics of *Cladophora* and coexisting filamentous algae in relation to environmental factors in freshwater ecosystems in Poland. Oceanol. Hydrobiol. Stud..

[5] Messyasz B., Pikosz M., Schroeder G., Łęska B., Fabrowska J., Kim S., Chojnacka K. (2015). Cultivation and Identification of Marine Algae. Marine Algae Extracts. Processes, Products, and Applications.

[6] Bourebaba L., Michalak I., Röcken M., Marycz K. (2019). *Cladophora glomerata* methanolic extract decreases oxidative stress and improves viability and mitochondrial potential in equine adipose derived mesenchymal stem cells (ASCs). Biomed. Pharmacother..

[7] Michalak I., Messyasz B. (2021). Concise review of *Cladophora* spp.: A macroalga of commercial interest. J. Appl. Phycol..

[8] Messyasz B., Łęska B., Fabrowska J., Pikosz M., Rój E., Cieslak A., Schroeder G. (2015). Biomass of freshwater *Cladophora* as a raw material for agriculture and the cosmetic industry. Open Chem..

[9] Michalak I., Tuhy Ł., Chojnacka K. (2015). Seaweed extract by microwave assisted extraction as plant growth biostimulant. Open Chem..

[10] Prazukin A.V., Anufriieva E.V., Shadrin N.V. (2020). Is biomass of filamentous green algae *Cladophora* spp. (*Chlorophyta*, *Ulvophyceae*) an unlimited cheap and valuable resource for medicine and pharmacology? A review. Rev. Aquac..

[11] Kasim W.A., Saad-Allah K.M., Hamouda M. (2016). Seed priming with extracts of two seaweeds alleviates the physiological and molecular impacts of salinity stress on radish (*Raphanus sativus*). Int. J. Agric. Biol..

[12] Ahmed D.A.E., Gheda S.F., Ismail G.A. (2021). Efficacy of two seaweeds dry mass in bioremediation of heavy metal polluted soil and growth of radish (*Raphanus sativus* L.) plant. Environ. Sci. Pollut. Res..

[13] Michalak I., Wilk R., Chojnacka K. (2017). Bioconversion of Baltic seaweeds into organic compost. Waste Biomass Valorization.

[14] Chekroun K.B., Baghour M. (2013). The role of algae in phytoremediation of heavy metals: A review. J. Mater. Environ. Sci..

[15] Bilal M., Rasheed T., Sosa-Hernández J.E., Raza A., Nabeel F., Iqbal H.M.N. (2018). Biosorption: An interplay between marine algae and potentially toxic elements—A review. Mar. Drugs.

[16] Bishnoi N.R., Kumar R., Kumar S., Rani S. (2007). Biosorption of Cr(III) from aqueous solution using algal biomass *Spirogyra* spp. J. Hazard. Mater..

[17] Ibrahim W.M. (2011). Biosorption of heavy metal ions from aqueous solution by red macroalgae. J. Hazard. Mater..

[18] Tamilselvan N., Saurav K., Kannabiran K. (2012). Biosorption of Cr (VI), Cr (III), Pb (II) and Cd (II) from aqueous solutions by *Sargassum wightii* and *Caulerpa racemosa* algal biomass. J. Ocean. Univ. China (Ocean. Coast. Sea Res.).

[19] Guarín-Romero J.R., Rodríguez-Estupiñán P., Giraldo L., Moreno-Piraján J.C. (2019). Simple and competitive adsorption study of nickel(II) and chromium(III) on the surface of the brown algae *Durvillaea antarctica* biomass. ACTS Omega.

[20] Hadadian M., Goharshadi E.K., Fard M.M., Ahmadzadeh H. (2018). Synergistic effect of graphene nanosheets and zinc oxide nanoparticles for effective adsorption of Ni (II) ions from aqueous solutions. Appl. Phys. A.

[21] Al-Qahtani K.M. (2017). Cadmium removal from aqueous solution by green synthesis zero valent silver nanoparticles with Benjamina leaves extract. Egypt. J. Aquat. Res..

[22] Azizi S., Shahri M.M., Mohamad R. (2017). Green synthesis of zinc oxide nanoparticles for enhanced adsorption of lead ions from aqueous solutions: Equilibrium, kinetic and thermodynamic studies. Molecule.

[23] Rangabhashiyam S., Balasubramanian P. (2018). Biosorption of hexavalent chromium and malachite green from aqueous effluents, using *Cladophora* sp. Chem. Ecol..

[24] Lee Y., Chang S. (2011). The biosorption of heavy metals from aqueous solution by *Spirogyra* and *Cladophora filamentous* macroalgae. Bioresour. Technol..

[25] Al-Homaidan A.A., Al-Qahtani H.S., Al-Ghanayem A.A., Ameen F., Ibraheem I.B.M. (2018). Potential use of green algae as a biosorbent for hexavalent chromium removal from aqueous solutions. Saudi J. Biol. Sci..

[26] Bulgariu L. (2020). Efficient use of algae biomass loaded with essential metal ions in the manufacture of feed additives. J. Appl. Phycol..

[27] Deng L., Su Y., Su H., Wang X., Zhu X. (2006). Biosorption of copper (II) and lead (II) from aqueous solutions by nonliving green algae *Cladophora fascicularis*: Equilibrium, kinetics and environmental effects. Adsorption.

[28] Deng L., Zhu X., Wang X., Su Y., Su H. (2007). Biosorption of copper (II) from aqueous solutions by green alga *Cladophora fascicularis*. Biodegradation.

[29] Tuzen M., Sari A. (2010). Biosorption of selenium from aqueous solution by green algae (*Cladophora hutchinsiae*) biomass: Equilibrium, thermodynamic and kinetic studies. Chem. Eng. J..

[30] Bağda E., Tuzen M., Sari A. (2017). Equilibrium, thermodynamic and kinetic investigations for biosorption of uranium with green algae (*Cladophora hutchinsiae*). J. Environ. Radioact..

[31] Michalak M., Baśladyńska S., Mokrzycki J., Rutkowski P. (2019). Biochar from a freshwater macroalga as a potential biosorbent for wastewater treatment. Water.

[32] Li R., Zhang T., Zhong H., Song W., Zhou Y., Yin X. (2021). Bioadsorbents from algae residues for heavy metal ions adsorption: Chemical modification, adsorption behaviour and mechanism. Environ. Technol..

[33] Skrzypczak D., Ligas B., Mikula K., Witek-Krowiak A., Samoraj M., Moustakas K., Chojnacka K. (2020). Valorization of post-extraction biomass residues as carriers of bioavailable micronutrients for plants and livestock. Biomass Conv. Bioref..

[34] Mohamed H.S., Soliman N.K., Abdelrheem D.A., Ramadan A.A., Elghandour A.H., Ahmed S.A. (2019). Adsorption of Cd^2+^ and Cr^3+^ ions from aqueous solutions by using residue of *Padina gymnospora* waste as promising low-cost adsorbent. Heliyon.

[35] Cardoso S.L., Costa C.S.D., Nishikawa E., Silva M.G.C., Vieira M.G.A. (2017). Biosorption of toxic metals using the alginate extraction residue from the brown algae *Sargassum filipendula* as a natural ion-exchanger. J. Clean. Prod..

[36] Venkatachalam P., Priyanka N., Manikandan K., Ganeshbabu I., Indiraarulselvi P., Geetha N., Muralikrishna K., Bhattacharya R.C., Tiwari M., Sharma N. (2017). Enhanced plant growth promoting role of phycomolecules coated zinc oxide nanoparticles with P supplementation in cotton (*Gossypium hirsutum* L.). Plant Physiol. Biochem..

[37] Nagarajan S., Arumugam Kuppusamy K. (2013). Extracellular synthesis of zinc oxide nanoparticle using seaweeds of gulf of Mannar. Indian J. Nanobiotechnol..

[38] Azizi S., Ahmad M.B., Namvar F., Mohamad R. (2014). Green biosynthesis and characterization of zinc oxide nanoparticles using brown marine macroalga *Sargassum muticum* aqueous extract. Mater. Lett..

[39] Namvar F., Rahman H.S., Mohamad R., Azizi S., Tahir P.M., Chartrand M.S., Yeap S.K. (2015). Cytotoxic effects of biosynthesized zinc oxide nanoparticles on murine cell lines. Evid. Based Complement. Alternat. Med..

[40] Ishwarya R., Vaseeharan B., Subbaiah S., Nazar A.K., Govindarajan M., Alharbi N.S., Kadaikunnan S., Khaled J.M., Al-anbr M.N. (2018). *Sargassum wightii*-synthesized ZnO nanoparticles—From antibacterial and insecticidal activity to immunostimulatory effects on the green tiger shrimp *Penaeus semisulcatus*. J. Photochem. Photobiol. B Biol..

[41] Priyadharshini R.I., Prasannaraj G., Geetha N., Venkatachalam P. (2014). Microwave-mediated extracellular synthesis of metallic silver and zinc oxide nanoparticles using macro-algae (*Gracilaria edulis*) extracts and its anticancer activity against human PC3 cell lines. Appl. Biochem. Biotechnol..

[42] Liu W., Zeb A., Lian J., Wu J., Xiong H., Tang J., Zheng S. (2020). Interactions of metal-based and metal-oxide-based nanoparticles (MBNPs and MONPs) with crop plants: A critical review of research progress and prospects. Environ. Rev..

[43] Wani A.H., Shah M.A. (2012). A unique and profound effect of MgO and ZnO nanoparticles on some plant pathogenic fungi. J. Appl. Pharmac. Sci..

[44] Mohamed M.M., Fouad S.A., Elshoky H.A., Mohammed G.M., Salaheldin T.A. (2017). Antibacterial effect of gold nanoparticles against *Corynebacterium pseudotuberculosis*. Int. J. Vet. Sci. Med..

[45] Amara U., Shad S., Ilyas N., Manaf A., Raja N.L., Mashwani Z.U.R. (2018). In vitro germination and biochemical profiling of Brassica napus in response to biosynthesised zinc nanoparticles. IET Nanobiotechnol..

[46] Sheela T., Arthoba Nayaka Y., Viswanatha R., Basavanna S., Venkatesha T.G. (2012). Kinetics and thermodynamics studies on the adsorption of Zn(II), Cd(II) and Hg(II) from aqueous solution using zinc oxide nanoparticles. Powder Technol..

[47] Somu P., Paul S. (2018). Casein based biogenic-synthesized zinc oxide nanoparticles simultaneously decontaminate heavy metals, dyes, and pathogenic microbes: A rational strategy for wastewater treatment. J. Chem. Technol. Biotechnol..

[48] Yogesh Kumar K., Muralidhara H.B., Arthoba Nayaka Y., Balasubramanyam J., Hanumanthappa H. (2013). Low-cost synthesis of metal oxide nanoparticles and their application in adsorption of commercial dye and heavy metal ion in aqueous solution. Powder Technol..

[49] Michalak I., Lewandowska S., Niemczyk K., Detyna J., Bujak H., Arik P., Bartniczak A. (2019). Germination of soybean seeds exposed to the static/alternating magnetic field and algal extract. Eng Life Sci..

[50] Yu Y., Navarro A.V., Sahuquillo À., Zhou G., López-Sánchez J.F. (2020). Arsenosugar standards extracted from algae: Isolation, characterization and use for identification and quantification purposes. J. Chromatogr. A..

[51] Fakhari S., Jamzad M., Fard H.K. (2019). Green synthesis of zinc oxide nanoparticles: A comparison. Green Chem. Lett. Rev..

[52] Elumalai K., Velmurugan S. (2015). Green synthesis, characterization and antimicrobial activities of zinc oxide nanoparticles from the leaf extract of *Azadirachta indica* (L.). Appl. Surf. Sci..

[53] Vaishnav J., Subha V., Kirubanandan S., Arulmozhi M., Renganathan S. (2017). Green synthesis of zinc oxide nanoparticles by *Celosia argentea* and its characterization. J. Optoelectron. Biomed. Mater..

[54] Itroutwar P.D., Govindaraju K., Tamilselvan S., Kannan M., Raja K., Subramanian K.S. (2020). Seaweed-based biogenic ZnO nanoparticles for improving agro-morphological characteristics of rice (*Oryza sativa* L.). J. Plant Growth Regul..

[55] Senthilkumar S.R., Thirumal S. (2014). Green tea (*Camellia sinensis*) mediated synthesis of zinc oxide (ZnO) nanoparticles and studies on their antimicrobial activities. Int. J. Pharm. Pharm. Sci..

[56] Gruyer N., Dorais M., Bastien C., Dassylva N., Triffault-Bouchet G. (2014). Interaction between silver nanoparticles and plant growth. Acta Hortic..

[57] Singh D., Kumar A. (2019). Assessment of toxic interaction of nano zinc oxide and nano copper oxide on germination of *Raphanus sativus* seeds. Environ. Monit. Assess..

[58] Mahmoud A.W.M., Abdelaziz S.M., El-Mogy M.M., Abdeldaym E.A. (2019). Effect of foliar ZnO and FeO nanoparticles application on growth and nutritional quality of red radish and assessment of their accumulation on human health. Agriculture.

[59] Rizwan M., Ali S., Ali B., Adrees M., Arshad M., Hussain A., Zia Ur Rehman M., Waris A. (2019). Zinc and iron oxide nanoparticles improved the plant growth and reduced the oxidative stress and cadmium concentration in wheat. Chemosphere.

[60] Rossi L., Fedenia L.N., Sharifan H., Ma X., Lombardini L. (2019). Effects of foliar application of zinc sulfate and zinc nanoparticles in coffee (*Coffea arabica* L.) plants. Plant Physiol. Biochem..

[61] Raskar S.V., Laware S.L. (2014). Effect of zinc oxide nanoparticles on cytology and seed germination in onion. Int. J. Curr. Microbiol. App. Sci..

[62] Taheri M., Qarache H.A., Qarache A.A., Yoosefi M. (2015). The effects of zinc-oxide nanoparticles on growth parameters of corn (SC704). STEM Fellowship J..

[63] Torabian S., Zahedi M., Khoshgoftarmanesh A. (2016). Effect of foliar spray of zinc oxide on some antioxidant enzymes activity of sunflower under salt stress. J. Agric. Sci. Technol..

[64] Afrayeem S.M., Chaurasia A.K. (2017). Effect of zinc oxide nanoparticles on seed germination and seed vigour in chilli (*Capsicum annuum* L.). J. Pharmacogn. Phytochem..

[65] Rajiv P., Vanathi P. (2018). Effect of parthenium based vermicompost and zinc oxide nanoparticles on growth and yield of *Arachis Hypogaea* L. in zinc deficient soil. Biocatal. Agric. Biotechnol..

[66] Rawat P.S., Kumar R., Ram P., Pandey P. (2018). Effect of nanoparticles on wheat seed germination and seedling growth. World Acad. Sci. Eng. Technol..

[67] Rai D., Moore D.A., Hess N.J., Rao L., Clark S.B. (2004). Chromium(III) hydroxide solubility in the aqueous Na^+^-OH^−^-H_2_PO_4_^−^-HPO_4_^2−^-PO_4_^3−^-H_2_O system: A thermodynamic model. J. Solution Chem..

[68] Hamdy A.A. (2000). Biosorption of heavy metals by marine algae. Curr. Microbiol..

[69] Murphy V., Hughes H., McLoughlin P. (2008). Comparative study of chromium biosorption by red, green and brown seaweed biomass. Chemosphere.

[70] Starmach K. (1975). Family: Cladophora Kutzing 1843. Identification Key. [Translation from: Flora Słodkowodna Polski 10, 227–263, 1972].

[71] Michalak I., Chojnacka K. (2010). The new application of biosorption properties of *Enteromorpha prolifera*. Appl. Biochem. Biotechnol..

[72] Ho Y. (2004). Citation review of Lagergren kinetic rate equation on adsorption reactions. Scientometrics.

[73] Yang J., Hou B., Wang J., Tian B., Bi J., Wang N., Li X., Huang X. (2019). Nanomaterials for the removal of heavy metals from wastewater. Nanomaterials.

[74] International Organization for Standardization (2005). ISO 14502-1:2005 Determination of Substances Characteristic of Green and Black Tea—Part 1: Content of Total Polyphenols in Tea—Colorimetric Method Using Folin-Ciocalteu Reagent.

[75] Brand-Williams W., Cuvelier M.E., Berset C. (1998). Use of a free radical method to evaluate antioxidant activity. LWT Food Sci. Technol..

[76] Ni Y., Chen S., Kokot S. (2002). Spectrophotometric determination of metal ions in electroplating solutions in the presence of EDTA with the aid of multivariate calibration and artificial neural networks. Anal. Chim. Acta.

